# Ultraviolet C inactivation of *Coxiella burnetii* for production of a structurally preserved whole cell vaccine antigen

**DOI:** 10.1186/s12866-024-03246-z

**Published:** 2024-04-04

**Authors:** Katja Mertens-Scholz, Amira A. Moawad, Elisabeth M. Liebler-Tenorio, Andrea Helming, Jennifer Andrack, Peter Miethe, Heinrich Neubauer, Mathias W. Pletz, Ina-Gabriele Richter

**Affiliations:** 1 Friedrich-Loeffler-Institut, Institute of Bacterial Infections and Zoonoses, Jena, Germany; 2 Institute for Infectious Diseases and Infection Control and Center for Sepsis Care and Control (CSCC), Jena University Hospital, Jena, Germany; 3 Friedrich-Loeffler-Institut, Institute of Molecular Pathogenesis, Jena, Germany; 4 Department of In Vitro Diagnostics Development, Research Centre of Medical Technology and Biotechnology, Erfurt, Germany; 5 Research Centre of Medical Technology and Biotechnology, Bad Langensalza, Germany

**Keywords:** UVC irradiation, *Coxiella burnetii*, Transmission electron microscopy, ELISA, Survival rate, Immunization, Antibody response

## Abstract

Q fever, a worldwide-occurring zoonotic disease, can cause economic losses for public and veterinary health systems. Vaccines are not yet available worldwide and currently under development. In this regard, it is important to produce a whole cell antigen, with preserved structural and antigenic properties and free of chemical modifications. Thus, inactivation of *Coxiella burnetii* with ultraviolet light C (UVC) was evaluated. *C. burnetii* Nine Mile phase I (NMI) and phase II (NMII) were exposed to decreasing intensities in a time-dependent manner and viability was tested by rescue cultivation in axenic medium or cell culture. Effects on the cell structure were visualized by transmission electron microscopy and antigenicity of UVC-treated NMI was studied by immunization of rabbits. NMI and NMII were inactivated at UVC intensities of 250 µW/cm^2^ for 5 min or 100 µW/cm^2^ for 20 min. Reactivation by DNA repair was considered to be unlikely. No morphological changes were observed directly after UVC inactivation by transmission electron microscopy, but severe swelling and membrane degradation of bacteria with increasing severity occurred after 24 and 48 h. Immunization of rabbits resulted in a pronounced antibody response. UVC inactivation of *C. burnetii* resulted in a structural preserved, safe whole cell antigen and might be useful as antigen for diagnostic purposes or as vaccine candidate.

## Introduction

*Coxiella burnetii*, a Gram-negative, obligate intracellular bacterium, is the etiological agent of the worldwide distributed zoonosis Q fever [1]. This disease is endemic in ruminants in Europe [2] and especially small ruminants are linked to human Q fever cases [3–5]. In animals, infections are often subclinical or cause late term abortions, weak offspring and fertility problems with a considerable economic impact [6–8]. The bacteria are shed within milk, urine, feces and especially in high amounts within birth products [9–11]. *C. burnetii* displays resistance to environmental stress and survives on sheep wool, in dry milk powder or in the soil for several months [12–14]. This is often associated with spore-like small cell variants (SCV). But data supporting this assumption are missing. The large cell variant (LCV) is metabolic active and replicates in a parasitophorous vacuole with phagolysosomal characteristics [15, 16]. Transmission to humans occurs through inhalation of contaminated aerosols, e.g. dust. Acute infection manifests in 40% of cases as a self-limiting flu-like illness presenting with fever and respiratory symptoms [17]. In 1–5% of cases the infection becomes chronic, which can be life-threatening [18, 19]. The financial burden of Q fever for public and veterinary public health was recently demonstrated for the largest ever reported Q fever outbreak in the Netherlands with over 4000 human cases and 74 Q fever related deaths [20, 21]. Intervention costs in the agricultural sector were estimated as 35,000€ per disability-adjusted life years (DALY) [20].

Vaccines and diagnostic test kits for humans and animals are currently based on formaldehyde inactivated bacteria. Use of vaccines has limitations and there is only one licensed vaccine for humans: Q-VAX (CSL Seqirus Australia Pty Ltd., Victoria, Australia), which is available in Australia only. It provides protection for a long time, but with a risk of severe side effects in pre-exposed persons [22]. Reports of Q-VAX failures exist for patients already infected during administration of the vaccine or patients which became ill after vaccination [23]. In Europe vaccination of cattle, goats and sheep is done with COXEVAC (Ceva Tiergesundheit GmbH, Düsseldorf, Germany) based on formaldehyde inactivated *C. burnetii* Nine Mile bacteria. Vaccination reduces shedding but can cause swelling at the injection site, a transient temperature increase and decrease in milk production in goats and cattle [24–26]. Swelling is more pronounced in infected animals [27]. Vaccination results in sheep are controversially discussed. One study reported no significant difference in bacterial shedding between vaccinated and non-vaccinated animals, whereas others reported a reduction of shedding [28, 29]. There is a strong need to develop new vaccines which confer protection and have limited side effects.

Veterinary diagnostic kits for antibody detection using formaldehyde inactivated Coxiellae as antigen were reported to have significantly variable specificities and sensitivities [30–32]. Contrary to human diagnostic assays, antibodies directed against the two antigenic variants of *C. burnetii*, termed phase I (smooth, full length LPS) and phase II (rough, truncated LPS) are not distinguished. Phase-specific antibodies are used in human diagnostics to differentiate between onset, acute and chronic Q fever [33]. This seems to be important for veterinary medicine also e.g. to identify chronic local infection of the udder and ongoing shedding in milk [34].

Formaldehyde inactivation is well-established and has been used for several decades for various pathogens. Known problems of this procedure are e.g. crosslinking of immunogenic structures that can lead to epitope loss or gain of new structures. This can reduce the efficiency of the vaccine and may lead to insufficient protection against subsequent infection [35]. This problem has been described for several pathogens [36, 37]. The most prominent example was the 1960ies vaccine against the respiratory syncytial virus (RSV), in which formalin inactivation has resulted in severe antibody dependent disease enhancement in infants [38, 39]. Therefore, the development of novel inactivation methods that are faster and better for antigen preservation are urgently needed. The germicidal activity of ultraviolet light C (UVC) is well established and might present an alternative to chemical inactivation. It causes mutagenic events by exciting electrons in DNA molecules and formation of pyrimidine dimers. These dimers cause replication errors and accumulation of mutations which may be lethal to the treated microorganism [40, 41]. Moreover, UVC generates reactive oxygen species (ROS) and sub-sequential oxidative damage to structural lipids and inhibits protein synthesis. These damages interfere with vital biological functions of bacteria [41]. The effectiveness of ultraviolet light (UV) inactivated vaccines was demonstrated for SARS coronavirus in mice and for vaccinia virus in macaques [42, 43]. However, many bacteria display the ability of reactivation. A process mediated by DNA repair mechanisms such as nucleotide excision repair (NER), base excision repair (BER) and homologous recombination, designated as dark repair. As a second line of defense, bacteria can express photolyases which directly rearrange bonds in a visible light depending manner [44]. This photoreactivation or light repair is more efficient than dark repair but both processes depend on adequate conditions [45, 46].

Thus, UVC exposure may be a promising method for inactivation of *C. burnetii*. The present study aims to determine the ability to inactivate axenic propagated *C. burnetii* Nine Mile phase I (NMI) and Nine Mile phase II (NMII) while preserving their antigenic structures. Thus, an antigen free of host cell materials can be gained, which is problematic to obtain for obligate intracellular bacteria.

## Materials and methods

### Bacterial strains and growth conditions

*Coxiella burnetii* Nine Mile RSA 493 phase I (NMI) and Nine Mile RSA 439 phase II (NMII) were propagated under biosafety level 3 and 2, respectively, in acidified citrate cysteine medium (ACCM-2, Sunrise Science Products, Knoxville, TN, USA) at 37 °C with 5% CO_2_ and 2.5% O_2_ [47]. Briefly, ACCM-2 was inoculated with 1 × 10^5^ genome equivalents per milliliter (GE/ml) and incubated for 7–10 days. Bacteria were harvested by centrifugation at 10.000 x *g* for 20 min at 4 °C and re-suspended in sucrose-glycerol solution (270 mM sucrose, 10% (v/v) glycerol) for preservation at -80 °C.

### Quantification of *Coxiella burnetii* by real-time PCR

*Coxiella burnetii* was quantified by using real-time PCR (qPCR) targeting the isocitrate dehydrogenase encoding gene *icd* [48, 49]. *C. burnetii* DNA was extracted by resuspending bacteria in phosphate-buffered saline (PBS) (BioWhittaker, Lonza, Walkersville, MD, USA) pH 7.4 and heat inactivated at 110 °C for 15 min. All qPCR reactions were performed in a 25 µl reaction mix using Maxima Probe/ROX qPCR Master Mix (ThermoFisher Scientific, Waltham, MA, USA), 300 nM each primer (icd-439 F and icd-514R), 100 nM probe (icd-464TM) and 2 µl of plasmid standard or sample [49]. The qPCR was carried out on a Mx3000P QPCR Instrument (Agilent Technologies, Santa Clara, CA, USA) using the following cycling conditions: 2 min at 50 °C, 10 min at 95 °C, followed by 45 cycles of 15 s at 95 °C and 30 s at 60 °C. Data collection and analysis was carried out using the Mx Pro4 software.

### UVC exposure of Coxiella burnetii

To determine the required UVC intensity for total growth inhibition, initial experiments were performed with *C. burnetii* NMII. Bacterial suspensions were adjusted to 1 × 10^10^ GE/ml in PBS pH 7.4 and for each experiment 3 ml aliquots were exposed in 6 well plates. The suspension had a filling height of 3 mm. UVC intensity was measured prior to exposure with a UVC solarmeter with a range of 0-1999 µW/cm^2^ (Solarmeter, Model 8.0, Glenside, PA, USA) and temperature monitored every 10 min with an Escort Junior device (Escort Messtechnik AG, Aesch bei Birmensdorf, Swizerland). NMII was exposed to different intensities, starting from 1500; 1000; 500; 250 to 100 µW/cm^2^ for 60; 45; 30; 20; 15; 10 and 5 min under agitation every 10 minutes. Depending on the results obtained for NMII, UVC intensities of 100 µW/cm^2^ and 250 µW/cm^2^ were chosen in a time dependent manner for total growth inhibition of NMI. Results represent the average of three independent experiments with three technical replicates each.

### Viability testing of *Coxiella burnetii* in ACCM-2 medium

Viability of *Coxiella burnetii* after UVC treatment was assessed after cultivation in axenic media and compared to an untreated control by qPCR. UVC treated bacteria were serially diluted from 1:10 to 1:10000. The untreated growth control was diluted to 10^5^ GE/ml in ACCM-2 in duplicate. From each dilution, two aliquots of 1 ml were harvested (16.000x *g*, 5 min, 4 °C) and resuspended in 200 µl PBS for quantification after heat inactivation (d0). After incubation for 7 days at 37 °C under 5% CO_2_ and 2.5% O_2_, bacterial cultures were harvested (16.000 x *g*, 5 min, 4 °C) and resuspended in 200 µl PBS for qPCR quantification after heat inactivation (d7). Viable bacteria were indicated by an increase of GE/ml on d7 compared to d0.

### Determination of the surviving fraction

The surviving fraction (S/S_0_) was determined as the quotient of colony counts (cfu/ml) from UVC treated at 250 µW/cm^2^, at 100 µW/cm^2^ and untreated bacteria and determined using a modified soft agarose overlay method as described by Omsland et al. [47]. Briefly, 200 µl of bacteria from serial dilutions in 2x concentrated ACCM-2 were mixed with 200 µl of 1% (w/v) melted ultrapure agarose (ThermoFisher Scientific, Waltham, MA, USA) at 60 °C and then equilibrated to 37 °C. From each dilution 100 µl drops in triplicate were applied on a solidified ACCM-2 agarose base. Plates were incubated for up to 10 days and colonies counted.

### Reactivation of *Coxiella burnetii* NMII in cell culture

Reactivation of *Coxiella burnetii* NMII treated with UVC was assessed by inoculation of buffalo green monkey (BGM) cells (Collection of Cell Lines in Veterinary Medicine; Friedrich-Loeffler-Institut, Greifswald Insel - Riems, Germany) and compared to an untreated control by visual assessment of *Coxiella*-containing vacuole (CCV) formation and qPCR. Bacteria were UVC treated as described above, incubated for 1 h in visible light and further incubated for 24 and 48 h in the dark at 37 °C at 2.5% O_2_ and 5% CO_2_. Samples were taken before, 1 h, 24 and 48 h after UVC exposure for inoculation of BGM cells (10^5^/ml, UltraMDCK medium, Lonza Walkersville, Inc.; Walkersville, USA) and cfu/ml determination. At each time point treated and untreated bacteria were serially diluted (undiluted; 1:10 till 10^–11^) and 50 µl aliquots added to BGM cells in 96 well plates. Inoculated cells were incubated for seven days and medium was changed once after 24 h incubation at 37 °C with 5% CO_2_. CCV formation was assessed by microscopy and cell layers harvested by scraping. Bacterial load was measured by qPCR after heat inactivation. For cfu/ml determination, 5 µl aliquots in tetraplicates of undiluted and serially diluted bacteria at each time point were dropped onto ACCM-2 agarose plates and incubated for 7 to 10 days. Results represent the average of three independent experiments with three technical replicates each.

### Transmission electron microscopy (TEM) analysis of UVC treated *Coxiella burnetii*

Triplicates of bacterial suspensions collected 1 h prior to UVC exposure, 1 h, 24 and 48 h after UVC exposure as well as 24 and 48 h without UVC exposure were fixed 1:2 (v:v) with 2.5% glutaraldehyde in 0.1 M cacodylate buffer (pH 7.2) containing 1.8% glucose for 1 h at 4 °C. The fixative was replaced by cacodylate buffer. Suspensions were vortexed for 5 min and an aliquot of 100 µl was removed from each suspension for negative staining.

For negative staining, drops of 30 µl were placed on a plate of dental wax. 300-mesh copper grids that had been filmed with formvar, coated with carbon and hydrophilized by glow discharge were floated on the drops for 30 min. The grids were briefly rinsed in 3 drops of distilled water and the excess liquid drained on wet filter paper. Finally, one grid of each preparation was contrasted on a drop of 0.5% and one on a drop of 0.2% uranyl acetate for 1 min. The excess contrast medium was drained on wet filter paper. After air-drying, grids were examined by transmission electron microscopy (Tecnai 12, FEI Deutschland Gmbh, Dreieich, Germany) at 80 kV. Representative micrographs were taken with a digital camera (TEMCAM FX416, TVIPS, Gauting, Germany) from five different quadrants of each grid at 2900x and 6800x magnification. The diameter of 10 bacteria of each preparation, which equals 30 bacteria of each treatment, were measured using the EM-Measure software (TVIPS, Gauting, Germany). Mean and standard deviation of diameters were calculated with excel and the percentage of SCV and LCV was determined. For this, bacteria with a diameter ≤ 370 nm were counted as SCV and bacteria with a diameter  ≥ 400 nm were counted as LCV. Bacteria in a transition stage with a diameter between 371 and 399 nm were not included in the counts.

For the preparation of ultrathin sections, suspensions were centrifuged for 15 min at 20.000x *g* to obtain a bacteria pellet. The pellet was removed from the tube, evenly mixed with 20 µl of 2% molten agarose on a glass slide, allowed to cool and sectioned into 1 mm³ cubes. Cubes were post-fixed in 2% osmium tetroxide and embedded in Araldite Cy212. Relevant areas were selected in Toluidine-blue-stained semi-thin sections. Ultrathin Sect. (85 nm) were stained with uranyl acetate and lead citrate and examined by transmission electron microscopy (Tecnai 12; FEI/Thermo Fisher Scientific) at 80 kV. Representative electron micrographs were taken at magnifications of both 4800x as well as 6800x using a 4kx4k digital CMOS camera (TEMCAM FX416, TVIPS, Garching, Germany) to evaluate bacterial morphology.

### Immunization of SPF-rabbits with UVC inactivated NMI

Immunization of SPF-rabbits (Zimmermann-Kaninchen, ZIKA rabbits; BioGenes) was outsourced (Assurance no. F16-00178 (A5755-01) BioGenes GmbH, Berlin, Germany). After collection of pre-immune sera, both rabbits were immunized with 200 µl of 8.3 × 10^9^ GE/ml NMI for the first step and with 50 µl of 8.3 × 10^9^ GE/ml NMI every 14 days for four times and subsequently every 21 days for 2 times. After a rest period of about 8-month two final boost were carried out with 100 µl of 1,1 × 10^9^ GE/ml NMI. Final blood was taken four days after the last immunization.

### Detection of *Coxiella burnetii* NMI and NMII reactive antibodies using indirect ELISA

Rabbit polyclonal sera were tested in tetraplicate using an indirect ELISA method. Briefly, high binding 96 well plates (Sarstedt AG & Co. KG, Nümbrecht, Germany) were coated with 2.5 × 10^7^ GE/ml NMI or NMII in PBS overnight at 4 °C. Unbound bacterial suspension was discarded and unspecific binding sites blocked with 5% (w/v) skim milk (Carl Roth GmbH & Co. KG, Karlsruhe, Germany) for 1 h at 37 °C. Plates were washed three times with PBS buffer containing 0.05% Tween 20 (v/v, Carl Roth GmbH & Co. KG, Karlsruhe, Germany) prior adding of serum samples in 2-fold dilutions ranging from 1:2000 to 1:128000 in 5% skim milk overnight at 4 °C. As positive control a polyclonal serum from rabbits immunized with heat-inactivated whole cell lysates of *C. burnetii* NMI (1:2000, Davids Biotechnology GmbH, Regensburg, Germany) or as negative control a polyclonal serum from SPF-rabbits (1:2000, Kaneka Eurogentec S.A., Seraing, Belgium) were used. After washing, bound antibodies were detected by peroxidase-conjugated goat anti-rabbit antibodies (1:5000, Dianova GmbH, Hamburg, Germany). Substrate solution (SeramunBlau slow2 50, Seramun Diagnostica GmbH, Heidesee, Germany) was added and the reaction stopped after 30 min with 0.5 M H_2_SO_4_ (Merck KGAA, Darmstadt, Germany). The plates were analyzed spectrophotometrically at 450 nm versus 630 nm.

Rabbit polyclonal sera were diluted 1:20000 and tested using the indirect ELISA ID Screen Q Fever Indirect (ID.vet, Innovative Diagnostics, Grabels, France) in duplicate. The secondary antibody was replaced by peroxidase-conjugated goat anti-rabbit antibodies (1:5000). The ELISA results were evaluated according to the manufacturer’s guidelines. Briefly, sera with a sample to positive ratio (S/P%) ≤ 40% was considered negative, a S/P% of 40% < and ≤ 50% suspicious, a S/P% of > 50% ≤ 80% as positive or > 80% as strong positive.

### Statistical analysis

All data were analyzed using GraphPad Prism version 9.4.1 (GraphPad Software, LLC, San Diego, USA). Pairwise comparisons of bacterial quantifications or diameter measurements were performed using the Kruskal-Wallis test due to invalid normality determined by the Shapiro-Wilk test. Data are presented as Tukey box plot with mean, quartiles Q_1_ and Q_3_. Statistically significant differences were indicated by p-values of < 0.05 (*), < 0.01 (**), and < 0.001 (***).

## Results

### Axenic propagated *Coxiella burnetii* NMI and NMII are effectively inactivated by UVC exposure

**Fig. 1:**
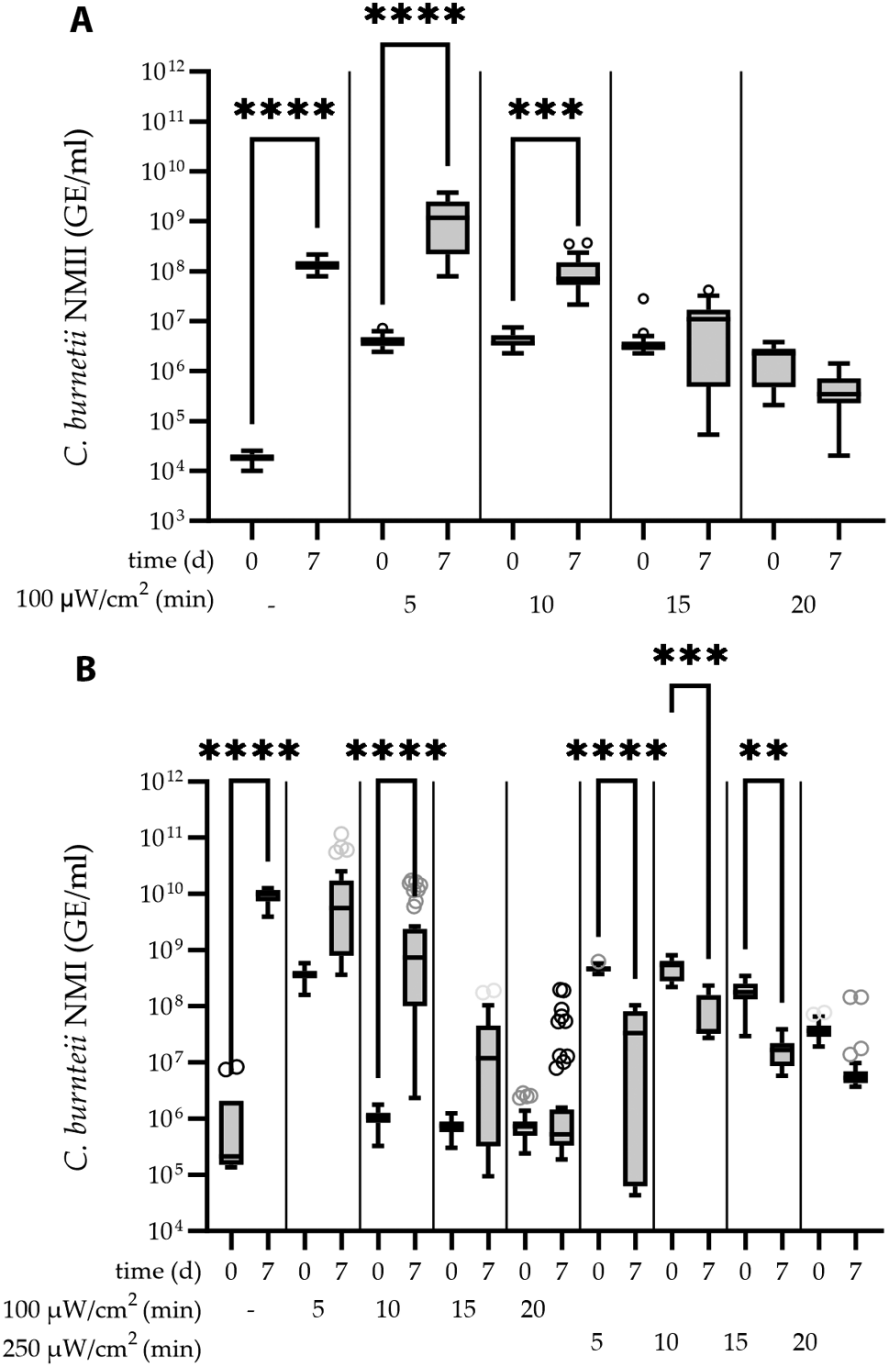
UVC treatment of (**A**) *C. burnetii* NMII by exposure to 100 µW/cm^2^ and (**B**) *C. burnetii* NMI to 100 µW/cm^2^ and 250 µW/cm^2^ in a time dependent manner. Bacteria (1 × 10^10^ GE/ml) were treated with UVC and rescued in ACCM-2. A decrease in recovered GE/ml at day 7 (d7) compared to day 0 (d0) indicates inactivation. Untreated bacteria were diluted to 1 × 10^5^ GE/ml and incubated for 7 days as growth control. The results presented as Tukey box plots represent the average of three independent experiments. Statistically significant differences are indicated by p-values of < 0.05 (*), < 0.01 (**), and < 0.001 (***)

The effective range for UVC treatment was established with NMII. An exposure to UVC light from 1500 µW/cm^2^ to 500 µW/cm^2^ for less than 15 min resulted in complete growth reduction of NMII as shown in Table 1. The genome equivalents per ml (GE/ml) quantified by realtime PCR (qPCR) dropped from the initial 1 × 10^10^ GE/ml of the bacterial solution prepared for treatment to 7.03 × 10^4^ GE/ml after exposure to 1500 µW/cm^2^ for 60 min or to 3,80 × 10^7^ GE/ml with 500 µW/cm*2* for 15 min. This indicates severe crosslinking of *Coxiella*-DNA due to UVC treatment. No increase of GE/ml, determined by qPCR, was observed after rescue cultivation in liquid ACCM-2 for 7 days. This indicates complete growth reduction of NMII at all intensities tested. Temperature monitoring could exclude a considerable effect of heat. The highest temperature of 21.7 ^o^C was recorded using 1500 µW/cm^2^ for 60 min. However, no increase in GE/ml was measurable by comparing the results of qPCR quantified bacteria from day 0 with day 7 at any intensity above 500 µW/cm^2^ (Table 1). At a lower intensity of 100 µW/cm^2^ bacteria could be rescued after 5 min, 10 and 15 min indicated by the significant increase in GE/ml after incubation for 7 days in ACCM-2 medium, but not after 20 min of exposure (Fig. 1A).

Growth reduction of *C. burnetii* NMI was evaluated accordingly. Bacteria did not grow after exposure to UVC with 250 µW/cm^2^ for 5 min as indicated by the significant decrease in GE/ml. At a lower UVC intensity of 100 µW/cm^2^ surviving bacteria could be rescued in liquid ACCM-2 after treatment for 5 min, 10 min, 15 and 20 min (Table 1; Fig. 1B).

**Table 1:** Growth of *Coxiella burnetii* NMI and NMII after UVC exposure

*C. burnetii* isolate	UVC Intensity (µW/cm^2^)	Exposure time (min)	*C. burnetii* (GE/ml (SD))^1^		Log difference^2^ day0/day7
			day 0	day 7	
NMII	1500	60	7.03E + 04 (8.77E + 04)	2.01E + 03 (8.48E + 02)	-1.54
	1000	60	2.54E + 06 (2.08E + 06)	1.01E + 05 (1.62E + 05)	-1.40
		15	5.08E + 06 (1.01E + 06)	1.90E + 05 (9.11E + 04)	-1.43
	500	60	1.09E + 07 (3.80E + 06)	1.29E + 05 (2.56E + 04)	-1.93
		45	2.22E + 07 (5.99E + 06)	3.77E + 05 (1.60E + 05)	-1.77
		30	2.74E + 07 (9.48E + 06)	9.83E + 05 (3.27E + 05)	-1.45
		15	3.80E + 07 (6.07E + 06)	3.25E + 06 (1.96E + 06)	-1.07
	250	15	7.23e + 05 (4.25e + 05)	2.05e + 05 (1.17e + 04)	-0.55
	100	20	2.24E + 06 (7.34E + 05)	9.48E + 05 (3.36E + 05)	-0.37
		15	5.85E + 05 (2.17E + 05)	1.77E + 06 (4.10E + 05)	0.48
		10	3.34E + 06 (4.03E + 05)	5,97E + 07 (8.87E + 06)	1.25
		5	3.27E + 06 (1.51E + 06)	1.76E + 08 (5.89E + 07)	1.73
NMI	250	15	1.57E + 08 (4.56E + 07)	2.72E + 07 (5.41E + 06)	-0.76
		10	4.73E + 08 (7.18E + 07)	3.43E + 07 (2.79E + 06)	-1.14
		5	1,80E + 08 (2,95E + 07)	3,45E + 07 (9,33E + 06)	-0.72
	100	20	6.27E + 05 (1.93E + 05)	1.17E + 07 (2.30E + 07)	1.27
		15	8.76E + 05 (1.64E + 05)	2.60E + 07 (1.36E + 07)	1.47
		10	1.14E + 06 (1.26E + 05)	8.76E + 07 (1.56E + 07)	1.89
		5	2.82E + 08 (8.51E + 07)	4.88E + 08 (9.23E + 07)	0.24

^1^For recovery, bacteria (1 × 10^10^ GE/ml) were diluted in ACCM-2 after UVC treatment (day 0) and incubated for 7 days

^2^Growth or inactivation was determined as log difference of qPCR quantified bacteria (GE/ml) after incubation. Results represent the average of three independent experiments

**Fig. 2:**
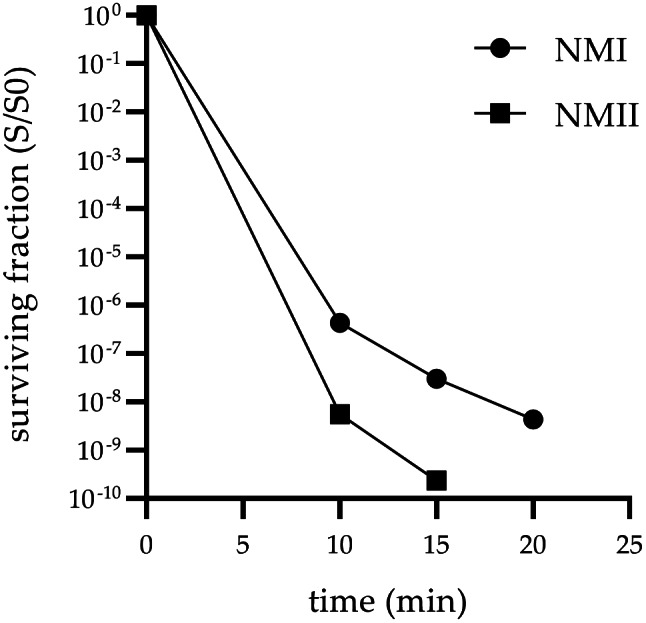
Surviving fraction of NMI and NMII after UVC exposure at 100 µW/cm^2^ for 0; 10; 15 and 20 min. Surviving fraction was determined as (S/S0) using colony forming units (cfu/ml) of UVC treated (S) and untreated (S0) bacteria. Results represent the average of three independent experiments

These results were confirmed by determination of the surviving fraction (cfu/ml). NMII bacteria were rescued after treatment at a UVC intensity of 100 µW/cm^2^ for 5 min, 10 and 15 min, whereas no colony forming units were detectable in suspensions treated for 20 min. The surviving fraction decreased accordingly with increasing exposure time (Fig. 2). Similar to NMII, the surviving fraction of NMI decreased with increasing exposure time at 100 µW/cm^2^ but without complete inactivation (Fig. 2). Taken together, growth of NMI and NMII is effectively inhibited by exposure to UVC but with different intensities and exposure times, respectively.

### *Coxiella burnetii* NMII does not reactivate after UVC treatment

UVC exposed bacteria were rescued after 1 h, 24 or 48 h in buffalo green monkey (BGM) cells or plated for cfu/ml counts. Independently from the given reactivation time no growth of UVC treated NMII was detectable in BGM cells. The bacterial load decreased accordingly to the serial dilution of the inoculum and no CCV formation was visible (Table 2). At all given reactivation times, the bacterial load in BGM cells after 7 days incubation decreased from approximately 1E + 05 to 1E + 03 accordingly to the dilution row. No bacteria were detectable in higher dilutions. In comparison, untreated NMII reached equal bacterial loads in BGM cells independently from the dilution of the inoculum after 7 days. CCV formation was visible in all dilutions till 10E-07 (Table 2). For UVC treated bacteria no cfu/ml could be determined. For the untreated control 2.47E + 08 cfu/ml could be detected, similar to the original suspension adjusted to 1E + 10 GE/ml by qPCR.

**Table 2:** Reactivation of UVC treated *C. burnetii* NMII

^1^Time after UVC exposure (h)	cfu/ml (SD)	^2^*C. burnetii* (GE/ml) in BGM cells				
		undiluted	1:10	1:100	1:1000	^3^Dilution with CCV formation
Untreated	2.47E + 08 (9.07E + 07)	1.62E + 07 (8.95E + 06)	6.97E + 06 (4.81E + 06)	6.59E + 06 (2.87E + 06)	4.06E + 06 (2.02E + 06)	undiluted to 1E-07
1	0	6.31E + 05 (1,61E + 05)	7.10E + 04 (1.63E + 04)	9.90E + 03 (1.34E + 03)	n.d.	n.d.
24	0	4.84E + 05 (4.86E + 04)	5.05E + 04 (7.77E + 03)	1.64E + 04 (2.25E + 04)	n.d.	n.d.
48	0	5.63E + 05 (3.85E + 05)	3.14E + 04 (2.53E + 03)	3.48E + 03 (8.39E + 02)	n.d.	n.d.

^1^*C. burnetii* NMII content was determined before (untreated) and at 1 h, 24 or 48 h after UVC treatment at 100 µW/cm^2^ for 20 min

^2^Bacteria were serially diluted prior inoculation of BGM cells and bacterial load was determined by qPCR detecting viable and dead bacteria after incubation for seven days

^3^CCV were detected by microscopy, wells with at least two clearly visible CCVs were considered positive.n.d., not detected

### UVC induced morphological changes

Negative contrast preparations and ultrathin sections were used to assess UVC induced morphological changes of *C. burnetii* NMII in a time dependent manner. Negative contrast preparation allowed to determine size and shape of Coxiella without section bias. Measurements focused on the bacterial diameter, since prior investigations had shown that large cell variants (LCV) and small cell variants (SCV) differed mainly in diameter, but not in length (personal communication).

**Fig. 3:**
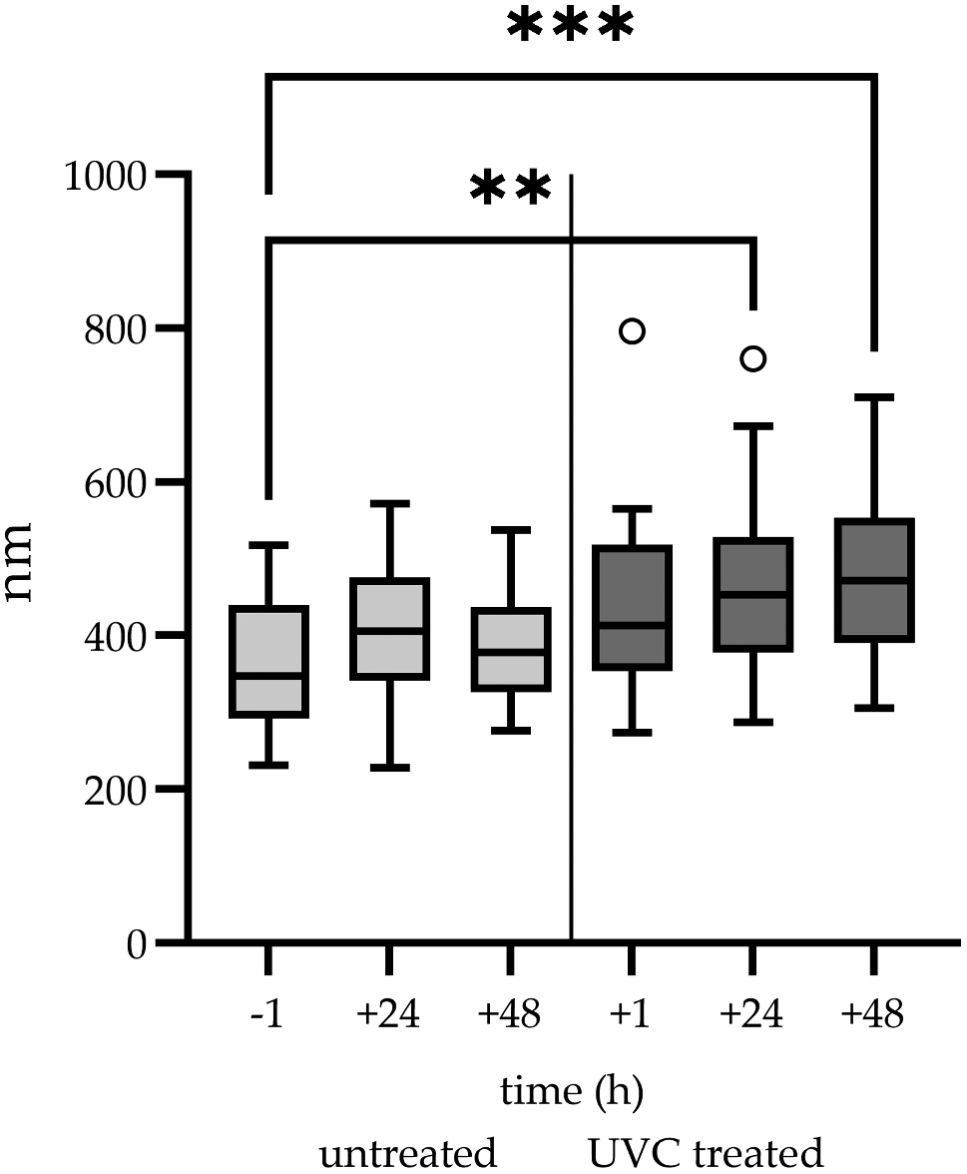
Effect of UVC treatment on the bacterial shape of *C. burnetii* NMII. Changes of the bacterial diameter (nm) are displayed for NMII bacteria before UVC treatment (-1), after 24 h (+ 24) and 48 h (+ 48) incubation as well as for UVC treated NMII 1 h (+ 1), 24 h (+ 24) and 48 h (+ 48) after UVC exposure at 100 µW/cm^2^ for 20 min. The results presented as Tukey box plots represent the average of three independent experiments. Statistically significant differences are indicated by p-values of < 0.05 (*), < 0.01 (**), and < 0.001 (***)

**Fig. 4:**
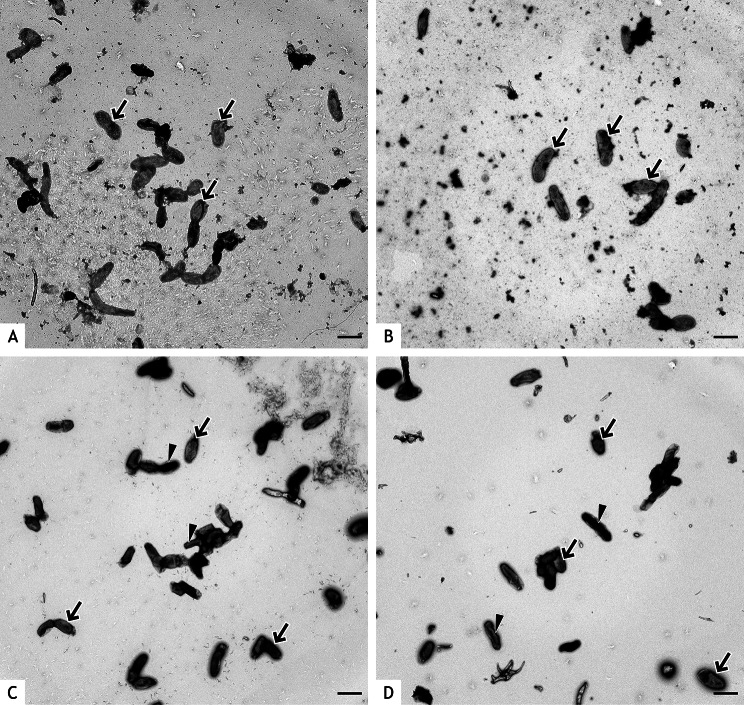
Negative contrast preparations of UVC treated and untreated *C. burnetii* NMII. Bacteria were UVC treated at 100 µW/cm^2^ for 20 min and negative contrast preparations were done after 24 h (**A**) and 48 h (**B**) incubation. Untreated controls were prepared accordingly after 24 h (**C**) and 48 h (**D**). Most *C. burnetii* treated with UVC (**A**, **B**) have large diameters resembling LCV (arrows indicate examples). Small pleomorphic particles representing break-down products of degraded bacteria are present at 24 h after UVC treatment (**A**) and increased at 48 h after UVC treatment (**B**). In the untreated controls (**C, D**), thick LCV (arrows, examples) and thin SCV (arrowheads, examples) can be distinguished at 24 and 48 h. Bars = 1 μm

The average diameter of *C. burnetii* NMII was lowest prior to UVC treatment, increased mildly within 24 h and decreased after 48 h (Fig. 3). This was associated with an increase of LCV at 24 h and a decrease at 48 h (Table 3) and may reflect the developmental cycle of *C. burnetii*. After UVC treatment, the average diameter of bacteria was higher compared to the untreated control and further increased significantly at 24 h and at 48 h after UVC treatment (Fig. 3). The comparatively high standard deviation in all UVC treated samples was the result of both, a few thin and a few exceedingly thick bacteria in these preparations. Based on diameter, high percentages of LCV were present after UVC treatment (Table 3). Bacteria with a large diameter were particularly electron lucent. An additional feature was the moderate amount of cellular detritus at 24 h and the large amount of cellular detritus at 48 h after UVC treatment indicating increased degradation of bacteria (Fig. 4A, B). In contrast, almost no detritus was present in the untreated controls at 24 and 48 h (Fig. 4C and D).

**Table 3:** Distribution of *C. burnetii* NMII morphological forms in untreated and UVC treated suspensions

Treatment	Time before or after treatment (h)	Number of bacteria measured (n)	^2^*C. burnetii* NMII morphological forms (%)	
			SCV	LCV
none	-1	27	70	30
	+ 24	27	41	59
	+ 48	23	52	48
^1^UVC	+ 1	26	35	65
	+ 24	24	21	79
	+ 48	27	18	82

^1^*C. burnetii* NMII was untreated or UVC treated at 100 µW/cm^2^ for 20 min and incubated for 1 h, 24 or 48 h

^2^*C. burnetii* small cell variants (SCV) and large cell variants (LCV) were distinguished based on diameter (nm)

**Fig. 5:**
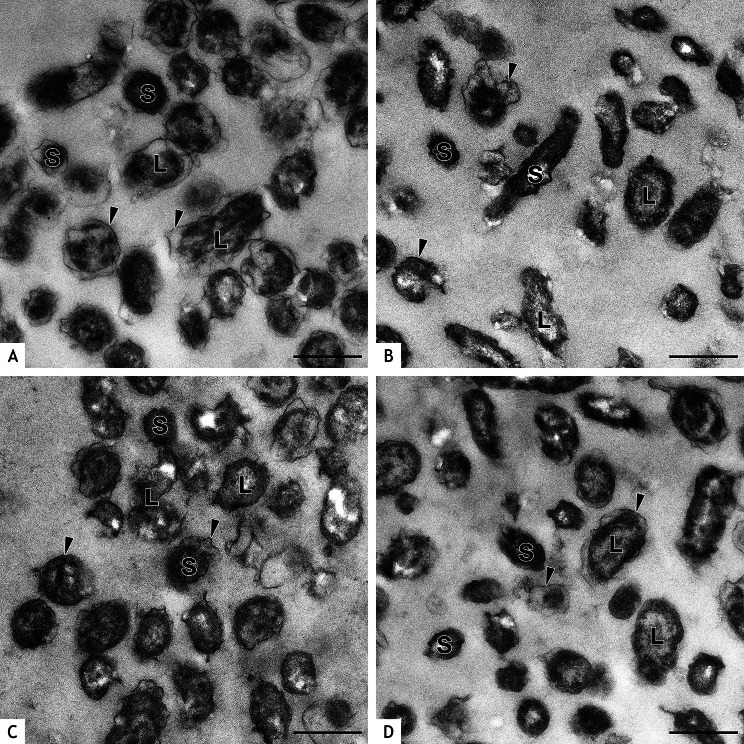
Ultrathin sections of *C. burnetii* NMII and NMI directly after UVC treatment. UVC treated (**A**) NMII at 100 µW/cm^2^ for 20 min and (**B**) NMI at 250 µW/cm^2^ for 5 min as well as untreated controls of (**C**) NMII and (**D**) NMI and were fixed for preparation of ultrathin sections within 60 min after exposure. Large cell variants (LCV; L, examples) and small cell variants (SCV; S, examples) are present in all bacterial suspensions. Distinct bacterial walls (arrowheads, examples) are clearly visible in the majority of Coxiella. There is no visible damage to the cell wall or cytoplasm and all bacteria appear undamaged. Bars = 500 nm

**Fig. 6:**
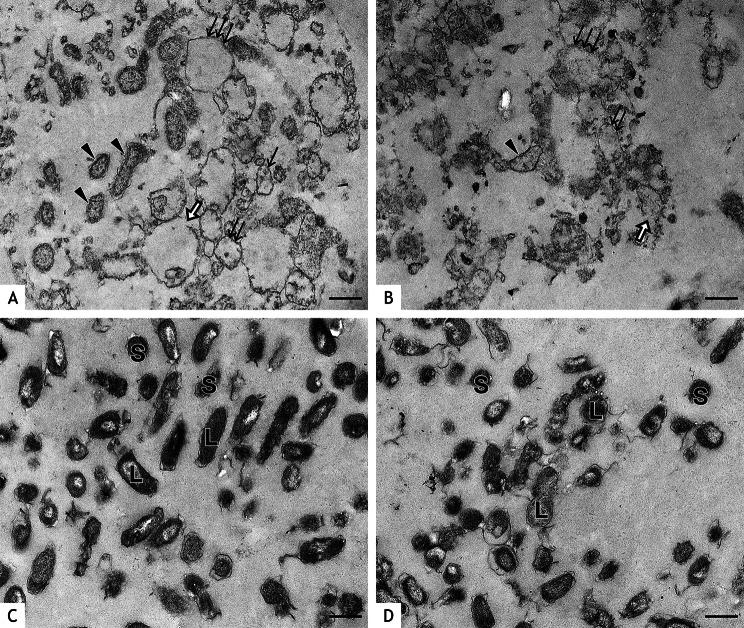
Ultrathin section of *C. burnetii* NMII after UVC treatment. *C. burnetii* NMII (**A)** 24 h and (**B**) 48 h after UVC treatment at 100 µW/cm^2^ for 20 min and untreated controls (**C**) after 24 h and (**D**) 48 h. Swelling and degradation of *Coxiella* is prominent 24 and 48 h after UVC treatment (**A, B**). Thin bacteria with coarsely granular electron lucent cytoplasm (arrowheads, examples) were interpreted as swollen SCV. Different degrees of swelling occurs in bacteria where the bacterial wall encloses lysed cytoplasm ( ↑ = mild, ↑↑ = moderate, ↑↑↑ = severe). Bacterial walls are occasionally fragmented (open arrows). LCV (L, examples) and SCV (S, examples) without visible damage are present in the untreated controls (**C, D**). Bars = 500 nm

Ultrathin sections revealed no morphological changes neither in *C. burnetii* NMI nor in NMII when bacteria where fixed within 60 min after UVC treatment (Fig. 5). General swelling of bacteria was observed at 24 and 48 h after UVC treatment (Fig. 6A, B). Discrimination of SCV and LCV was no longer possible because of the lysis of cytoplasm. These findings indicate that most of the bacteria identified as LCV in the negative staining preparations were most likely swollen bacteria. Different degrees of swelling were identified ranging from comparatively thin bacteria (SCV) with coarsely granular electron lucent cytoplasm to increasingly distended bacteria where only bacterial walls were left and the cytoplasm was completely dissolved. Some of these bacterial walls were intact, others were fragmented. Membrane fragments formed small vesicles. There were no marked differences in the findings between 24 and 48 h after treatment. In the time-matched untreated controls, all bacteria had cytoplasm of higher electron density, and LCV and SCV could be differentiated (Fig. 6C, D).

### Antibody response after immunization with UVC inactivated *Coxiella burnetii* NMI

**Fig. 7:**
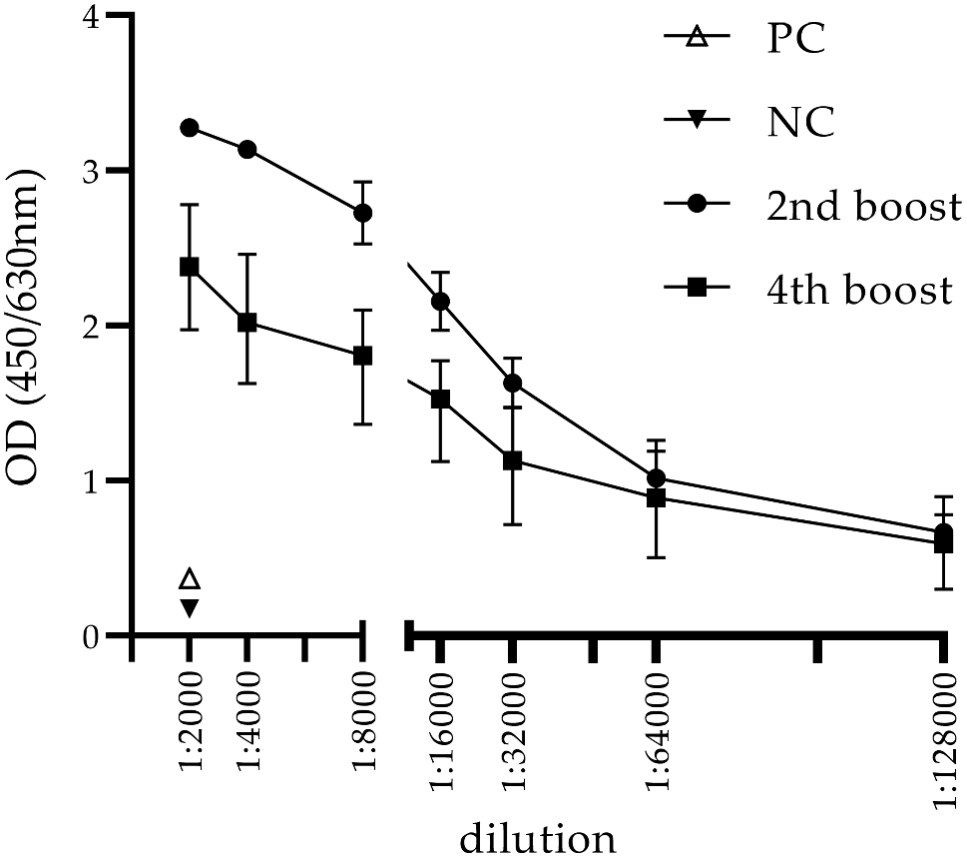
Reactivity of sera from two SPF-rabbits (Zimmermann Kaninchen, ZIKA) immunized with UVC inactivated *C. burnetii* NMI after the second and fourth booster. Plates were coated with 2.5 × 107 GE/ml of NMI. Serum samples from two rabbits were serially diluted and traced with a horseradish-conjugated goat anti-rabbit antibody. As positive control (PC) a polyclonal rabbit serum against heat-inactivated whole cell lysate of *C. burnetii* NMI and as negative control (NC) a polyclonal SPF-rabbit serum was used. Results represent the average of two independent experiments

The antibody titer of two SPF-rabbits immunized with UVC inactivated *C. burnetii* NMI was analyzed by titration against viable *C. burnetii* NMI. Pre-immune sera of both rabbits were tested and resulted in an OD_450_ value below 0.04. Titers of both rabbits were above 1:128000 after the second and fourth booster immunization (Fig. 7). Prediluted rabbit sera (1:20000) collected after the fourth booster immunization were tested strong positive with the commercial ID Screen Q Fever Indirect ELISA Test Kit with a S/P% of 94.5 and 94.3, respectively. This indicates that the antigenic properties of UVC inactivated *C. burnetii* are intact since generated antibodies react with untreated *C. burnetii* NMI as well as with a bovine derived *C. burnetii* antigen presented in the used commercial ELISA.

## Discussion

The effect of UVC exposure on the viability of *Coxiella burnetii* was studied in the early 1950ties, but those results are difficult to interpret. Ransom and Huber (1951) reported inactivation of *C. burnetii* yolk sac preparations after 0.3 s exposure to a Levinson-Oppenheimer lamp of unknown intensity [50]. Another study reported that dried Coxiella survived exposure to a “bacterial lamp” with 30 W at 75 cm distance to the sample [51]. Also exposure of *C. burnetii*-suspensions prepared from spleens of mice exposed to UV at 25 W in 1 m distance was not effective and viable bacteria were detected by inoculation of mice [52]. In these studies, neither the quantity of bacteria used nor the applied intensities of light are specified. In a more recent study, 10^8^ NMI or NMII bacteria in suspension or cultured in macrophages were inactivated by UV treatment with 600 µW/cm^2^ at 10 cm distance for 15 s [53]. These results confirm the here reported data, that *C. burnetii* NMI and NMII are completely inactivated at any UVC intensity above 500 µW/cm^2^ likely due to extensive DNA damage. This was indicated by the significant decrease of genome equivalents after UVC treatment. Contrary to all studies mentioned above, a UVC solarmeter was used to determine the UVC intensity at sample level. A complete inactivation was achieved even at low intensities of 250 µW/cm^2^ and 100 µW/cm^2^ for suspensions of both, the virulent strain NMI as well as the laboratory strain NMII.

UVC has a low penetration depth and is quenched by turbid or protein-containing liquids [54, 55]. The suspensions used in this study were prepared with PBS and had a filling height of 3 mm. Additionally, every 10 min the plates were agitated for mixing. Despite these precautions for equal exposure of the bacteria to UVC, complete inactivation should be always tested carefully for each batch, since bacteria display the ability to reverse UVC induced DNA damage by DNA repair mechanisms and start to replicate again. *C. burnetii* has an efficient DNA repair system which is constitutively expressed [56]. Further it encodes for a photolyase (*phrB*, CBU_1176, UniProt Q83CE4) of 38% amino acid sequence identity to that of *Escherichia coli* (*phrB*, UniProt P00914). Photoreactivation is the most efficient repair system and depends on light and environmental factors such as temperature or salinity [46]. It was shown to rescue *E. coli* after 99.9% UVC inactivation but effectivity is highly variable in different strains [57, 58]. Photo repair activity is measurable in less than 20 min after UVC treatment. Therefore, reactivation was investigated 1 h, 24 and 48 h after UVC exposure by determination of cfu/ml, bacterial loads in BGM cells by qPCR or CCV formation [46]. The lack of *C. burnetii* growth indicated that there was no reactivation.

The activity of repair mechanisms might have been influenced by the fact that a suspension of *C. burnetii* in PBS was exposed to UVC and this environment does not resemble the interior of the *Coxiella*-containing vacuole (CCV). Further experiments using axenic media or cell culture for assessment of enzymatic activity need to be carried out to examine if photo repair or dark repair are active mechanisms and play a role after UVC treatment for rescue of intracellular *C. burnetii*. The dependence of *C. burnetii* on the pH gradient between the lumen of the CCV and the bacterial cytoplasm was discovered a long time ago and lead to the designation of metabolic acid activation. The gradient is utilized for transport and utilization of nutrients and generation of ATP using proton motive force [59–61]. Medium with slightly neutral or neutral pH leads to decreased metabolic activity and reduced replication [62]. This pH gradient is not present in PBS used for preparation of bacterial suspension and may have additionally reduced the ability of *C. burnetii* to repair UVC induced damage on the DNA and protein level.

UVC exposure leads to rearrangements of the phospholipid bilayer, pore formation, causes lipid oxidation and induces changes in the amount and composition of fatty acids [63–66]. This is not immediately reflected in the cellular structure resulting in morphologically intact LCV and SCV directly after UVC treatment. This allows to obtain structurally intact antigen of inactivated Coxiellae. Severe effects of UVC exposure on the morphology of Coxiellae became visible at 24 h after exposure which explains the inactivation. They were characterized by swelling of the bacteria, rarefaction of cytoplasm and finally fragmentation of the bacterial cell wall. The swelling made a discrimination between LCV and SCV impossible. The high number of LCV identified in the negative contrast preparation most likely represent swollen bacteria and are not an indication of changes in the *C. burnetii* life cycle. Swelling is a fundamental expression of acute cellular injury. Failure of energy dependent membrane pumps to control ion gradients and pore formation may result in an influx of water and dilution of cytoplasm. Similar morphological changes were reported for *Shigella flexneri* after UVC treatment [67]. The different degrees of swelling observed may also reflect differences in the reaction of LCV and SCV which differ in membrane structure [68]. There was no indication of recovery at 48 h after UVC exposure which further supports the absence of reactivation.

The necessary UVC intensity was higher for total growth inhibition of NMI than for NMII. The only difference between these two isogenic strains is the severely truncated lipopolysaccharide (LPS) in NMII [69, 70]. The LPS is the dominant component of the outer leaflet of the outer membrane (OM) and plays an important role as barrier against antibiotics or host defense factors. It stabilizes the OM due to the interaction of the O-polysaccharide side chains and binding of divalent cations. Bacteria with a deep rough LPS accumulate phospholipid patches which result in higher membrane fluidity and higher permeability [71, 72]. It is known that UVC exposure produces reactive oxygen species which attack nucleic acids and proteins. Additionally, UVC leads to lipid oxidation and pore formation [66, 73]. Interestingly, the amount of free endotoxin increases upon UV irradiation of *Escherichia coli* [74]. This might indicate that the LPS confers some kind of protection against UVC exposure for NMI and NMII is more sensitive for irradiation.

Bacterial vaccines are often produced by chemical inactivation with ß-propiolactone or formaldehyde [75]. Despite several effective vaccines based on formaldehyde inactivated pathogens e.g. poliovirus or cholera, negative effects of the crosslinking nature of formaldehyde on effectiveness of the preparations are known. Induced structural and antigenic changes may cause a lack of specific epitopes or generation of new unspecific epitopes and thus have a decreased efficiency [36, 76, 77]. An alternative strategy for gentle inactivation is low-energy electron irradiation leaving the bacterial cell structure intact which has been proven for bacterial and viral pathogens [78, 79]. UVC inactivation of pathogens provides a rapid and low-cost method when vaccines are urgently demanded, e.g. during the SARS-CoV pandemic. This technique is feasible in many settings. The generated antigen contains all relevant structures such as quaternary structures and glycosylation of proteins relevant for immunogenicity. The UVC derived *C. burnetii* NMI antigen resulted in a pronounced antibody response in rabbits. Furthermore, these rabbit sera reacted strongly with viable NMI and NMII as well as in a commercial ELISA kit for detection of *C. burnetii*-specific antibodies in sera from ruminants. This implies that UVC inactivated NMI bacteria display highly similar antigenic properties as viable bacteria. To confirm this, the antigenic nature of UVC treated bacteria needs to be analyzed in more detail. Similar results for immunization with UVC inactivated pathogens were reported for SARS-CoV virus. Immunized mice produced a strong IgG response with neutralizing activities [43, 80]. Application of UV inactivated vaccinia virus elicits a strong antibody response in macaques and reduced the viral load [42]. Taken together, the antibody response in SPF rabbits imply that UVC inactivated bacteria may confer protection but this needs to be evaluated in further studies.

## Conclusions

UVC treatment is suitable for inactivation of virulent *C. burnetii* in translucent liquids under laboratory settings. After each treatment the effective inactivation has to be confirmed for safety reasons. The structural integrity of the NMI bacteria is preserved at 250 µW/cm2 for 5 min. This antigen is safe and free of host cell materials originating from classical propagation of *C. burnetii*. It provokes a strong antibody response in rabbits and might be suitable for the development of new serological tests or as a component of a vaccine.

## Data Availability

The datasets supporting the conclusions of this article are available in the Zenodo repository after publication, DOI: 10.5281/zenodo.10217973.
